# Patient-Specific Sedation Management via Deep Reinforcement Learning

**DOI:** 10.3389/fdgth.2021.608893

**Published:** 2021-03-31

**Authors:** Niloufar Eghbali, Tuka Alhanai, Mohammad M. Ghassemi

**Affiliations:** ^1^Human Augmentation and Artificial Intelligence Laboratory, Department of Computer Science, Michigan State University, East Lansing, MI, United States; ^2^Laboratory for Computer-Human Intelligence, Division of Engineering, New York University Abu Dhabi, Abu Dhabi, United Arab Emirates

**Keywords:** medication dosing, personalized medicine, deep reinforcement learning, propofol, sedation management

## Abstract

**Introduction:** Developing reliable medication dosing guidelines is challenging because individual dose–response relationships are mitigated by both static (e. g., demographic) and dynamic factors (e.g., kidney function). In recent years, several data-driven medication dosing models have been proposed for sedatives, but these approaches have been limited in their ability to assess interindividual differences and compute individualized doses.

**Objective:** The primary objective of this study is to develop an individualized framework for sedative–hypnotics dosing.

**Method:** Using publicly available data (1,757 patients) from the MIMIC IV intensive care unit database, we developed a sedation management agent using deep reinforcement learning. More specifically, we modeled the sedative dosing problem as a Markov Decision Process and developed an RL agent based on a deep deterministic policy gradient approach with a prioritized experience replay buffer to find the optimal policy. We assessed our method's ability to jointly learn an optimal personalized policy for propofol and fentanyl, which are among commonly prescribed sedative–hypnotics for intensive care unit sedation. We compared our model's medication performance against the recorded behavior of clinicians on unseen data.

**Results:** Experimental results demonstrate that our proposed model would assist clinicians in making the right decision based on patients' evolving clinical phenotype. The RL agent was 8% better at managing sedation and 26% better at managing mean arterial compared to the clinicians' policy; a two-sample *t*-test validated that these performance improvements were statistically significant (*p* < 0.05).

**Conclusion:** The results validate that our model had better performance in maintaining control variables within their target range, thereby jointly maintaining patients' health conditions and managing their sedation.

## Introduction

Intensive care units (ICUs) serve patients with severe health issues who need continuous medical care and monitoring (1). In the course of their treatment within ICUs, patients generate a wide variety of data that are stored in electronic health record systems including computed tomography scans, care-provider free-text notes, clinician treatment decisions, and patient demographics. The task of a clinician is to carefully consider these data to infer the latent disease *state* of their patients and (given this state) apply an optimal treatment *policy* (a set of *actions*) that will maximize the odds of short-term patient survival and longer-term patient recovery. This sequential inference process used by clinicians during care is one instance of a greater class of problems referred to as reinforcement learning (RL) in the artificial intelligence community.

Interest in the applications of RL to healthcare has grown steadily over the last decade. Within the last few years, numerous works have demonstrated the potential of RL methods to help manage sensitive treatment decisions in sepsis (1–5), sedation regulation (6, 7), mechanical ventilation (1, 8), and medication dosing (9–11). Refer to the works of Liu and Prescott (12) and Yu et al. (1) for a recent systematic review of RL models in critical care and healthcare. In this article, we demonstrate the use of deep RL for the regulation of patient sedation. Sedation is essential for invasive therapies such as endotracheal intubation, ventilation, suction, and hemodialysis, all of which may result in patient pain or discomfort when conducted without the assistance of sedatives (13, 14); it follows that sedation management is an important component of effective patient treatment in critical care environments.

Sedation management is particularly challenging because ICU patients enter treatment for a variety of health reasons (often with incomplete medical records) and may require prolonged periods of sedation as they recover (15, 16). Overdosing sedatives has been associated with several negative health outcomes including longer recovery times, increased need for radiological evaluation, increased odds of long-term brain dysfunction, and death (7, 17). Conversely, underdosing sedatives may result in untreated pain, anxiety, and agitation, which have been associated with patient immunomodulation and posttraumatic stress disorder (13). Hence, great care must be taken in the delicate process of sedation management (14), where patients may exhibit unique pharmacological responses for the same dose of a given medication. This results in pharmacokinetic or pharmacodynamic variations for the same drug administered with the same frequency in different individuals (18, 19). In order to address this issue, a growing number of clinical studies have proposed automated methods based on patients' evolving clinical phenotypes to deliver safe and effective sedation regulation (6, 16, 20).

RL is a promising methodological framework for sedation regulation because it can learn nuanced dosing policies that consider variation in disease intensity, drug responsiveness, and personal patient characteristics (1, 20). In the past few decades, several RL-based models have been proposed to regulate sedation in the ICU (6, 7, 21–29). However, most sedation management methods exhibit one or more of the following limitations: (1) incomplete physiological context or patient response variability, (2) use of simulated data for validation, (3) failure to account for common clinical practices such as attempts to minimize the total dosage of sedatives (17), and (4) assumption of discrete state and action spaces resulting in sensitivities to heuristic choices of discretization levels (5). Lastly, most of the prior work has focused on a specific medication—propofol—which has no intrinsic analgesic effect and must be coadministered with an opioid or other analgesic for ICU patients (30).

Our work herein extends previous studies by employing an RL framework with continuous state-action spaces to identify an optimal dosing policy for *both* a common sedative and opioid medication *together* (propofol and fentanyl). Our proposed model considers interindividual differences to reach the target level of sedation as measured by the Riker Sedation–Agitation Scale (SAS), while also minimizing the total sedative amount administered. Although our sedation measure is based on patient behaviors, which do not directly reflect the brain, they are useful as an optimization target for both their reliability and ease of collection (31); the SAS is a progressive sedation–agitation indicator with excellent interrater reliability (32).

## Materials and Methods

In this section, the critical care data set and our preprocessing approach are introduced. The decision-making framework and its associated RL components are discussed afterward.

### Data

#### Database

All data for this study were collected from the Medical Information Mart for Intensive Care (MIMIC-IV), a freely accessible ICU data resource that contains de-identified data associated with more than 60,000 patients admitted to an ICU or the emergency department between 2008 and 2019 (33, 34).

#### Key Variables

We extracted 1,757 patients from MIMIC who received a commonly used sedative (propofol) and opioid (fentanyl) during their ICU stay; for each of these patients, we also extracted a time series of sedation level according to SAS. SAS is a 7-point ordinal scale that describes patient agitation: 1 indicates “unarousable,” 4 indicates “calm and cooperative,” and 7 indicates “dangerous agitation” levels. SAS serves as our therapeutic target for this work; it has been shown previously that optimization of patients' level of sedation is associated with decreased negative outcomes, such as time spent on mechanical ventilation (17). We note that our study population excluded all patients diagnosed with severe respiratory failure, intracranial hypertension, status epilepticus traumatic brain injury, acute respiratory distress syndrome, and severe acute brain injury (including severe traumatic brain injury, poor-grade subarachnoid hemorrhage, severe ischemic/hemorrhagic stroke, comatose cardiac arrest, status epilepticus) because sedation management approaches for such patients are idiosyncratic (35, 36).

#### Measures Utilized

According to the American Society of Anesthesiologists, current recommendations for monitoring sedation include blood pressure (diastolic blood pressure and mean noninvasive blood pressure), respiration rate, heart rate, and oxygen saturation pulse oximetry (Spo_2_) (37); we utilized these measures in our modeling approach. Additionally, we utilized measures based on studies conducted by Yu et al. (1) and Jagannatha et al. (38), including arterial pH, positive end-expiratory pressure (PEEP), inspired oxygen fraction (Fio_2_), arterial oxygen partial pressure, plateau pressure, average airway pressure, mean arterial pressure (MAP), age, and gender.

A total of 14 features were used to describe patients in our data: diastolic blood pressure, mean noninvasive blood pressure, respiration rate, heart rate, Spo_2_, arterial pH, PEEP, Fio_2_, arterial oxygen partial pressure, plateau pressure, average airway pressure, MAP, age, and gender (dichotomized, with male coded as 0). Prior to modeling, all continuous measures were zero-mean variance normalized.

Table 1 presents summary information about the final data set, which contained a total of 1,757 subjects, with a 100% survival rate, a mean age of 68.5 years, and a mean ICU stay of 149.8 h. Table 2 provides summary statistics of the measures based on different levels of sedation defined by SAS. The final row presents the proportion of data available in each level, which exhibits a Gaussian distribution with the mean at SAS level 4 out of 7 (calm and cooperative).

**Table 1:** Summary of data set.

**Gender**	**% Survivors**	**Mean age (y)**	**Mean hours in ICU**	**No. of patients**
**Female**	100	75	157	806
**Male**	100	65	146	1,301
**Total population**	**100**	**69**	**149**	**1,757**

**Table 2:** Summary statistics of selected features based on different levels of sedation [Riker Sedation–Agitation Scale (SAS)]. Last row presents the proportion of data in each level.

**Features\SAS**	**SAS = 1 Unarousable**	**SAS = 2Very sedated**	**SAS = 3 Sedated**	**SAS = 4Calm, cooperative**	**SAS = 5 Agitated**	**SAS = 6Very agitated**	**SAS = 7 Dangerous agitation**
Noninvasive blood pressure mean	74 ± 17	72 ± 16	74 ± 17	76 ± 71	79 ± 18	79 ± 19	81 ± 17
Diastolic blood pressure	59 ± 15	60 ± 19	60 ± 23	64 ± 418	69 ± 625	65 ± 18	66 ± 15
Heart rate	86 ± 21	88 ± 19	89 ± 477	88 ± 213	91 ± 21	94 ± 18	94 ± 19
Respiration rate	21 ± 7	21 ± 38	20 ± 8	20 ± 9	21 ± 6	21 ± 6	22 ± 7
Arterial PH	7 ± 0	7 ± 0	7 ± 0	7 ± 0	7 ± 0	7 ± 0	7 ± 0
Positive end-expiratory pressure set	7 ± 4	9 ± 5	7 ± 3	5 ± 3	5 ± 3	6 ± 3	6 ± 2
Oxygen saturation pulse oximetry (Spo_2_)	96 ± 7	96 ± 6	97 ± 5	97 ± 40	97 ± 3	96 ± 6	97 ± 3
Inspired oxygen fraction (Fio_2_)	52 ± 18	54 ± 17	47 ± 13	46 ± 70	46 ± 15	47 ± 16	55 ± 21
Arterial oxygen partial pressure	137 ± 69	126 ± 65	123 ± 57	120 ± 53	120 ± 58	122 ± 57	117 ± 44
Plateau pressure	21 ± 6	23 ± 8	20 ± 6	18 ± 4	19 ± 5	20 ± 6	19 ± 3
Average airway pressure	12 ± 5	14 ± 6	11 ± 12	7 ± 3	9 ± 13	9 ± 4	8 ± 3
Mean arterial pressure (MAP)	80 ± 20	79 ± 25	83 ± 74	88 ± 42	89 ± 41	100 ± 63	85 ± 29
**Proportion of data %**	**3.32**	**6.37**	**20.47**	**53.15**	**5.94**	**0.45**	**0.06**

#### Preprocessing and Time Windowing

For each patient, we divided the ICU stay duration into hourly contiguous windows. A given window may contain multiple recordings of a given measure. In windows with more than one recording, the mean of the recording was used. To address missing data, we removed entries where data for all measures, or the SAS outcome, were missing and applied the sample-and-hold interpolation technique. We imputed any remaining missing values with the mean value of the missing measure calculated across the training data.

#### Training, Validation, and Testing Set Partition

We partitioned our data at the subject level into a training (60%, 1,055 subjects, 156,303 time windows), validation (20%, 351 subjects, 49,997 time windows), and test set (20%, 351 subjects, 55,493 time windows). The training data set was used to identify model parameters; the validation set was used to identify model hyperparameters, and the testing set was used to evaluate the model's ability to generalize to data unseen during training.

### Model Architecture

The sedation dosing problem can be cast as a Markov Decision Process (MDP) where the purpose is to find an optimal dosing policy that, given the patient's state, specifies the most effective dosing action (1, 9). Our RL model is based on a deep deterministic policy gradient (DDPG) approach introduced by (39). DDPGs benefit from the advantages of deterministic policy gradients (DPGs) (40) and deep Q networks (41), which robustly solve problems in continuous action spaces. In order to learn the optimal policy, we used an *off-policy* RL algorithm that studied the success (and failures) of the clinicians' policies in our data set. In the following sections, the proposed method is elaborated.

#### Policy

We modeled the sedation management problem as an MDP described by the tuple (*S, A, P, R*), in which

- **s**_**t**_ ∈ *S* is the patient state containing the 14 dimensional feature vector described above in a given hourly window *t*;
- **a**_**t**_ ∈ *A* is a two-dimentional action vector corresponding to the quantity of propofol and fentanyl administered in a given hourly window.
- *P*(**s**_**t**+**1**_| *s*_*t*_, *a*_*t*_) is the probability of the next state vector given the current state vector and the action taken.
- *r*(**s**_**t**_, *a*_*t*_) ∈ *R* is the observed reward following a state transition at time window *t* that is related to how closely the SAS and blood pressure of the patient match the optimal value (discussed in *Reward*).

Given our formulation of the sedation management problem, we trained an RL agent that (1) observes the current patient state **s**_**t**_, (2) updates the medication doses with an optimal action **a**_**t**_, and (3) receives a corresponding reward *r*(**s**_**t**_, *a*_*t*_) before moving to the next state **s**_**t**+**1**_ and continuing the process. For the agent to maximize its cumulative reward over several state-action pairs, it must learn a policy π–a function that maps states (patient's state) to actions (drug dosages): *a* = π(*s*). In training, the RL agent uses a sequence of observed state-action pairs (*s*_*t*_, *a*_*t*_), called a trajectory (τ), to learn the optimal policy π^*^ by maximizing the following objective function:


(1) $$\text{J}\left(\pi \right)=𝔼\left[\text{R}(\tau )\right]={𝔼}_{\text{s}}[\int_{\text{a}}\text{p}(\tau |\pi )\text{R}(\tau )d\tau ]$$

where $\text{R}\left(\tau \right)={\text{r}}_{\text{t}}+\gamma {\text{r}}_{\text{t}+1}+\gamma^{2}{\text{r}}_{\text{t}+2}+\gamma^{3}{\text{r}}_{\text{t}+3}+\ldots +\gamma^{\text{T}}{\text{r}}_{\text{t}+\text{T}}$ is a sum of discounted rewards, γ is a discount factor that determines the relative weight of immediate vs. long-term rewards, and θ denotes the set of model parameters learned during RL training. If γ is close to 0, the agent is biased toward short-term rewards; if γ is close to 1, the agent is biased toward longer-term rewards. In our case, the value of γ was 1E-3 and was determined by exploring several values of γ and retaining the value that maximized the model's performance on the validation set.

**Figure 1:**
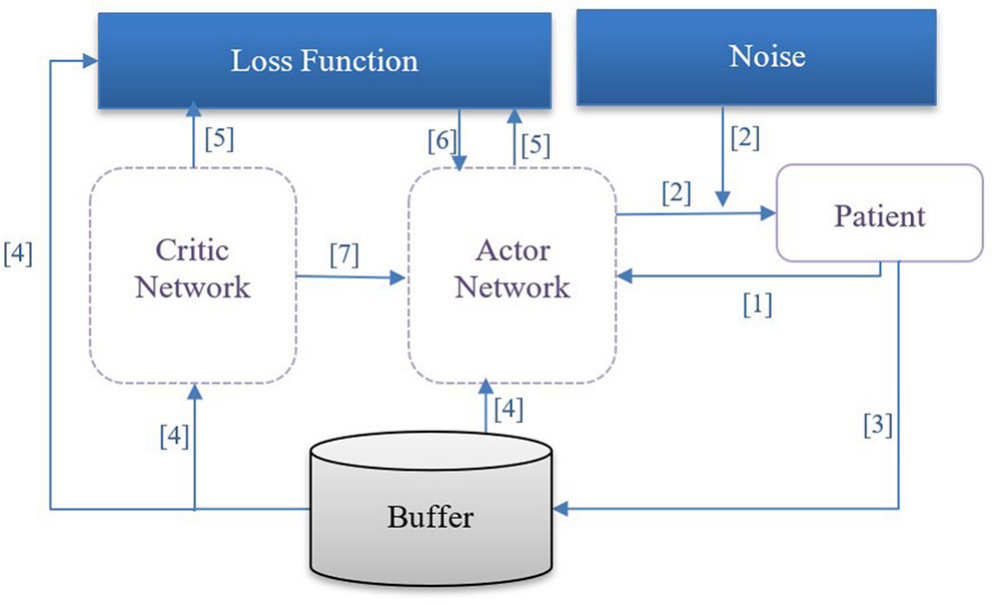
DDPG procedure: [1] The agent observes patient's state **s**_**t**_ and transfers it to the actor network. [2] The actor network receives **s**_**t**_ as an input and outputs the dosage amount plus a small noise (action); the purpose of the noise is to promote exploration of the action space. [3] The agent observes a reward **r**_**t**_, and patient's next clinical state **s**_**t**+**1**_; the tuple of < **s**_**t**_, **a**_**t**_, **r**_**t**_, **s**_**t**+**1**_> is retained in an experience pool. [4] From the experience pool, a batch of N tuples will be selected to learn the optimal policy. [5] The temporal difference loss function is computed. [6] The critic network is updated by minimizing the temporal difference loss. [7] The actor network is updated using the deterministic policy gradient theorem.

In our case, the specific formulation of π^*^ is determined via DDPG, which employs four neural networks to ultimately learn the optimal policy from the trajectories: a Q network (critic), a deterministic policy network (ac), a target Q network, and a target policy network. The “critic” estimates the value function, while the “actor” updates the policy distribution in the direction suggested by the critic (for example, with policy gradients). The target networks are time-delayed copies of their original networks that slowly track the learned networks and greatly improve the stability of learning. Similar to deep Q learning, DDPG utilizes a replay buffer to https://www.powerthesaurus.org/collect/synonymscollect experiences for updating neural network parameters. During each trajectory, all the experience tuples (state, action, reward, next state) will be stored in a finite-sized cache called “replay buffer.” At each time window, the actor and critic are updated by sampling a minibatch from the buffer. The replay buffer allows the algorithm to benefit from learning across a set of uncorrelated transitions. Instead of sampling experiences uniformly from replay buffer, we have used prioritized experience reply (42) to replay important transitions more frequently, thereby learning more efficiently. In our case, the next state **s**_**t**+**1**_, is computed by a neural network consisting of three fully connected layers with ReLu activation functions in the first two layers and a linear activation in the final layer. Batch normalization was used during training. Models were implemented in Pytorch 1.6.0 and used Adam optimization (43). We illustrate the procedure of DDPG for finding the optimal policy for medication dosing in Figure 1 and describe the procedure below:

- The agent observes the patient's state **s**_**t**_ and transfers it to the actor network.
- The actor network receives **s**_**t**_ as an input and outputs the dosage amounts plus a small noise (actions); the purpose of the noise is to promote exploration of the action space.
- The agent observes a reward **r**_**t**_ and the patient's next clinical state **s**_**t**+**1**_. The tuple of < **s**_**t**_, **a**_**t**_, **r**_**t**_, **s**_**t**+**1**_> is stored in a pool of experiences.
- From the pool of experiences, a batch of N tuples will be used to learn policies.
- The loss function [temporal difference (TD)] is then computed.
- The critic network is updated by minimizing the loss.
- The actor network is updated using the DDPG theorem.

#### Reward

In order to learn from the trajectories, our RL agent requires a formal definition of reward based on deviations from the control variables (SAS, MAP). Propofol administration lowers sympathetic tone and causes vasodilation, which may decrease preload and cardiac output and consequently lower the MAP and other interrelated hemodynamic parameters. Therefore, ensuring a desired range of MAP is an essential consideration of propofol infusion (7, 44). Moreover, efforts should be made to minimize the sedative dosage (17). Under these premises, the reward issued to the sedation management agent at each time window is defined with the purpose of keeping SAS and MAP measurements at the clinically acceptable and safe range while penalizing increases in dose; for our purposes, these ranges are described by the following equations:


(2) $${\text{r}}_{MAP}\;=\;\frac{2}{1+{\text{e}}^{-(MA{\text{P}}_{\text{t}}-65)}}-\frac{2}{1+{\text{e}}^{-(MA{\text{P}}_{\text{t}}-85)}}-1\;$$

(3) $${\text{r}}_{RSS}\;=\;\frac{2}{1+{\text{e}}^{-(SA{\text{S}}_{\text{t}}-3)}}-\frac{2}{1+{\text{e}}^{-(SA{\text{S}}_{\text{t}}-4)}}-1$$

where *r*_*MAP*_ assigns value close to 1 when MAP values fall within the therapeutic range of 65–85 mmHg and negative values elsewhere; *r*_*RSS*_ assigns value close to 1 when SAS value falls within the therapeutic range of 3–4 and negative values elsewhere. Target therapeutic ranges are selected based on Hughes et al. (17) and Padmanabhan et al. (7), respectively.

Next, let **D**_**t**_ describe deviations from the clinically acceptable and safe range of SAS and MAP in time window *t* with the static lower target boundary (LTB) and upper target boundary (UTB) described above:


(4) $${\text{D}}_{\text{t}}\left(\text{control variable}\right)\{\begin{array}{c} \begin{array}{cc} \text{if measured value for control variable is in target range },\; & 0\; \end{array}\\ \text{if measured value for control variable}<\text{LTB },\text{ LTB }-\text{measured value for control variable }\\ \text{if measured value for control variable}>\text{UTB },\text{ UTB }-measured\;value\;for\;control\;variable\; \end{array}$$

From this deviation, we may compute the total error in time window *t* from both control variables as follows:


(5) $$erro{\text{r}}_{\text{t}}\;=\;{\text{D}}_{\text{t}}\left(MAP\right)+{\text{D}}_{\text{t}}\left(SAS\right)$$

If **e**_**t**+**1**_ (deviation from target range for MAP and SAS at time window *t* + 1) is ≥ **e**_**t**_, then we assign **r**_**t**+**1**_ = 0, which serves to penalize a “bad” action.


(6) $${\text{r}}_{\text{t}}\;=\;\{\begin{array}{c} {\text{r}}_{SAS}+{\text{r}}_{MAP}-0.02\;{\text{r}}_{dosage\;if\;{\text{e}}_{\text{t}}<{\text{e}}_{\text{t}-1}}\\ 0\text{ otherwise } \end{array}$$

where r_dosage_ is the amount of the medications provided.

### Performance Evaluation Approach

We compared the performance of our model to the recorded performance of the clinical staff with the reasonable assumption that the clinical staff intended to keep patients within the therapeutic range during their ICU stay. For this purpose, the performance error is defined for each trajectory (hours spent in ICU) as follows:


(7) $$\begin{array}{l} PE_{i}^{c}=\frac{\text{patient }i\text{ ICU duration }-\text{ time control variable }c\text{ is in target range }}{\text{patient }i\text{ ICU duration}}\times 100 \end{array}$$

Equation 7 captures the proportion of the total ICU stay hours that patient *i* spent outside the therapeutic range for the control variable *c* ∈ {*SAS, MAP*}. If the measured value falls within the target interval, the difference between the measured value and the target value will be zero; otherwise, the difference will be computed based on the target interval boundaries. More specifically, to assess the sedation management performance of the trained agent against the clinical staff, the root mean square error (RMSE), mean performance error (MPE), and median performance error (MDPE) were compared for chosen actions under both our model policy and the clinicians' policy (24). MDPE gives the control bias observed for a single patient and is computed by:


(8) $${\text{MDPE}}_{\text{i}}^{\text{c}}=\text{median}\left({\text{PE}}_{\text{i}}^{\text{c}}\right)$$

${\text{RMSE}}_{\text{i}}^{\text{c}}$ is the RMSE for each patient and control variable, which is computed using


(9) $${\text{RMSE}}_{i}^{c}=\sqrt{\frac{\sum_{\text{t}=1}^{\text{N}}{\left({\text{D}}_{\text{t}}\left(c\right)\right)}^{2}}{N}}$$

where *N* represents ICU stay duration in hours, and *t* iterates over the set of hourly measurements for each patient *i*.

## Results

For assessment purposes, we applied our model to the held-out test set (351 patients, 55,493 h); patients in the test set had a mean ICU duration of 158 h.

In Table 3, we present the performance for both the learned sedation management policy and clinicians' policy (as reflected by the data). Table 3 indicates that MDPE and RMSE for our model are lower than that of clinicians; this means that our learned sedation management policy may reduce the amount of time a patient spends outside the therapeutic range when compared to the clinicians. As seen in Table 3, the measured values for SAS and MAP are within the target range for 91.3% and 82.2% of the patient ICU duration, respectively. These results correspond to a 26% (MAP) and 8% (SAS) improvement in MPE, compared to the clinicians' policy. A two-sample *t-*test validates that the reduction of performance error and RMSE in our model is significant (*p* < 0.05) compared to the clinicians' policy; the results validate that our model had better performance in maintaining control variables within their target range, thereby jointly maintaining patients' health condition and managing their sedation.

**Table 3:** Performance metrics for control variables SAS (Riker Sedation–Agitation Scale) and MAP (mean arterial pressure).

**Performance metric**	**Control variables**			
	**Learned policy**		**Clinician's policy**	
	**MAP**	**SAS**	**MAP**	**SAS**
MPE %	17.82 ± 9.22	8.69 ± 1.14	44.66 ± 23.18	17.43 ± 21.54
MDPE %	15.0	0	45.45	0.69
Mean RMSE	23.45	0.08	46.38	0.71
Mean Values	74.99 ± 4.47	3.42 ± 0.07	85.26 ± 28.4	3.47 ± 1.04
Mean propofol dosage	10.49 ± 60		24.23 ± 132	
Mean fentanyl dosage	15.9 ± 8.9		15.1 ± 2.3	

*The MPE (mean performance error), MDPE (median performance error), and RMSE (root mean square error) values for learned policy are lower for both control variables, which means our model had a better performance in keeping these variables in their target range*.

**Figure 2:**
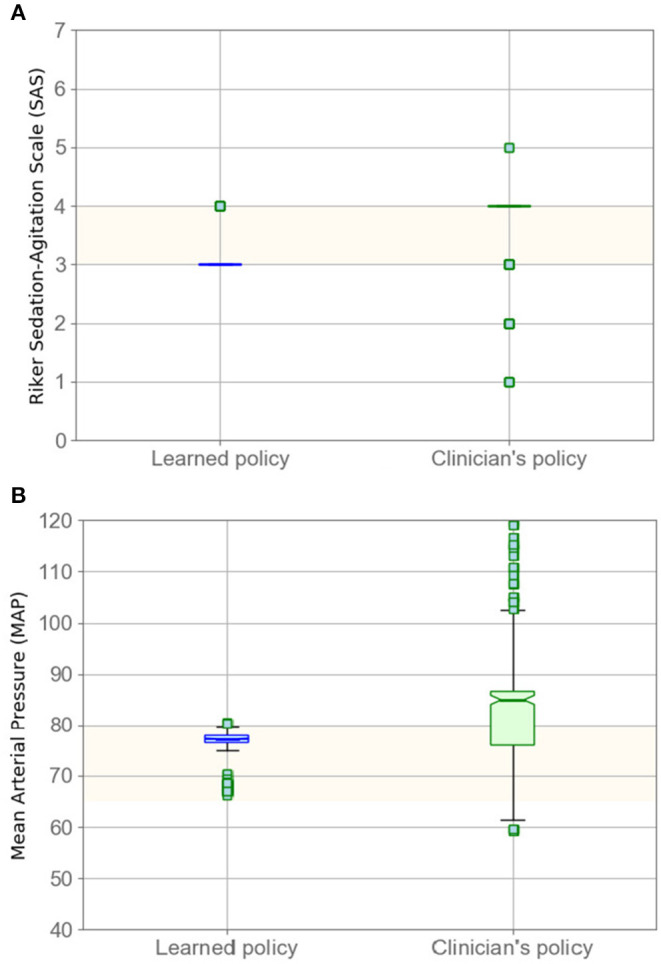
Evaluating policy in terms of SAS **(A)** and MAP **(B)** boxplots. Left boxplots (blue) correspond to our model, whereas the right boxplots are clinicians' results. Highlighted areas identify the target range.

In Figure 2, we compare the SAS and MAP value distributions using a boxplot; the green box corresponds to our model's results. The figure indicates that that our policy has promising results for sedation management while keeping MAP in the target range. The lower SAS values predicted by our model, as seen in Figure 2, are reasonable as our model suggests less medication, on average, which therefore leads to lower levels of sedation (lower SAS).

In Table 3, we show the mean medication amount for patients for both the learned policy and clinicians' policy. We assessed the ability of our model to lower the total amount of medication administered while maintaining the therapeutic status of patients. More specifically, for each patient trajectory, we computed the medication administered by our policy, compared to the clinicians. A two-sample *t-*test indicated a statistically significant reduction in the total amount of medication administered by our RL agent (*p* < 0.03) compared to the clinicians. Thus, we conclude that dosage amounts administered to patients following our model is lower than the clinician's prescription.

**Figure 3:**
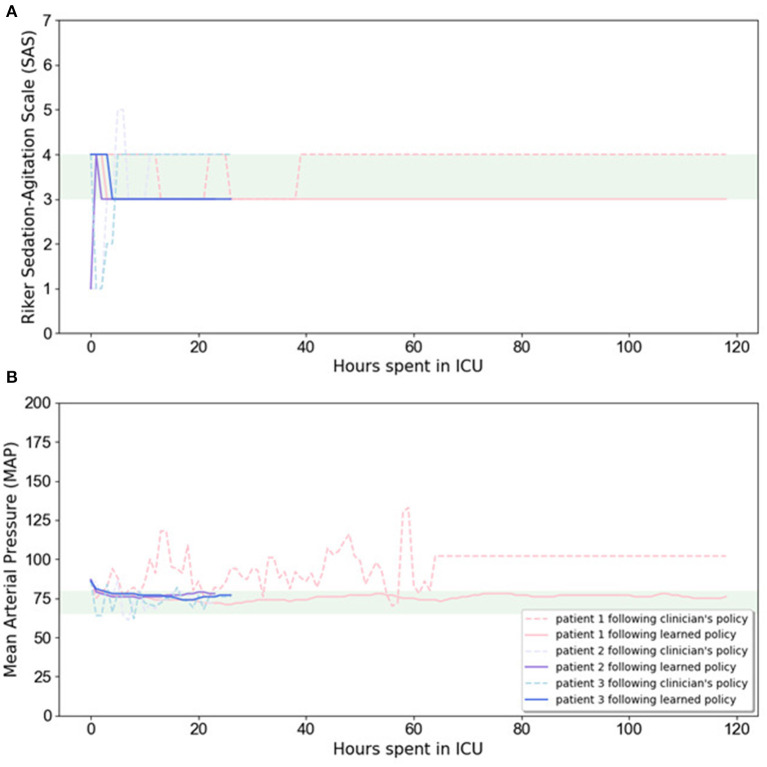
Variation in SAS **(A)** and MAP **(B)** values for three randomly selected patients during ICU stay. Dashed lines depict the changes when using clinician's policy, while constant lines are related to learned policy, and the highlighted area is the target range.

In Figure 3, we illustrate the RL-based closed-loop sedation scenario for three randomly selected patients. The figure shows the variation in SAS and MAP values for three randomly selected patients during ICU stay; dashed lines depict the changes when using the clinician's policy, constant lines represent our proposed policy, and the green area represents the target range. Figure 3 illustrates the ability of our model to drive SAS values to the therapeutic range without drastic deviation from the MAP therapeutic range for these three randomly selected patients. The evaluation results confirm that the RL agent is able to maintain the SAS value and MAP value in the target ranges while lowering the medication amount.

## Discussion

In this study, we proposed a deep RL method based on a DDPG approach to manage propofol administration while considering the dynamic observations that were available in patient's electronic medical records. We utilized RL because it is an effective framework for deriving optimal and adaptive regulation of sedatives for patients with different responses to the same medication and is able to learn an optimal sequence of decisions from retrospective data. Moreover, RL-based methods can be practically applied to real clinical practice by taking simple steps. RL has two main components: the *environment* (patient) and the *agent* (our sedative regulator). Every time the agent performs an action (recommends dosage), the patient gives a reward to the agent, which can be positive or negative depending on how appropriate the dosage was from that specific state of a patient. The goal of the agent is to learn what dosage maximizes the reward, given every possible state of the patient. *States* are the observations that the agent receives at each step in the patients' care process. Using retrospective data from medical records, our agent will learn from the set of patient states, administered dosage, response to the doses, and the reward it gets. After initial training of the agent, it is able to generalize over the state space to recommend doses in situations it has not previously encountered. In a practical setting, the state observed by the agent may be either extracted from the electronic medical record directly or provided by the clinician through a user interface.

This work extends previous studies in a number of ways. First, our trained agent operates in a continuous action space; this distinguishes it from prior models that utilized Q learning for medication dosing with an arbitrary discretization of the action space. Second, we used the SAS to assess the patient's sedation level, which is one of the most widely used sedation scales in the ICU, but instead of merely regulating sedation level, we also trained our agent to consider hemodynamic parameters (MAP) by reflecting them in the reward function. Third, in practical clinical settings, it is common to minimize the sedative dosage, which is unaccounted for in prior works on medication dosing using RL. To address this limitation, we penalized the increase in medication dosage while learning the optimal policy. Our test results confirm the ability of our model to manage sedation while also lowering the dosage in comparison to clinicians' prescriptions. Therefore, our policy leads to lower administration of sedatives in comparison to the clinicians' policy; the sedation level during sedative administration is close to the lower target SAS boundary, which corresponds to higher sedation.

Administration of sedatives such as propofol can have adverse effects on the hemodynamic stability of patients. Specifically, propofol causes vasodilation leading to a decrease in MAP (7). Our results indicate a notable improvement (26%) in MAP management compared to the recorded performance of clinicians. This achievement is important because if MAP drops below the therapeutic range for an extended period, end-organ manifestations such as ischemia and infarction can occur. If MAP drops significantly, blood will not perfuse cerebral tissues, which may result in loss of consciousness and anoxic injury (45).

We conclude that our sedation management agent is a promising step toward automating sedation in the ICU. Furthermore, our model parameters can be tuned to generalize to other commonly used sedatives in ICU and will work with other sedation monitoring scales such as bispectral index or Richmond Agitation and Sedation Scale.

Further efforts need to be taken in order for the method described herein to be effective enough for real-world deployment. Long-term anesthetic infusion often results in drug habituation, and hence, a patient's pharmacologic response may change over the course of their treatment (44); future approaches may need to account for the effects of habituation. Additionally, future work in this domain would benefit by accounting for other factors that confound sedation in the ICU environment including adjunct therapies such as clonidine, ketamine, volatile anesthetics, and neuromuscular blockers. We validated our model based on an assumption that clinicians were dosing patients with an intention to achieve the target sedation level (as defined by ICU protocols). However, this could be untrue in some cases; for example, some procedures performed in the ICU require a deeper sedation level, which contradicts our assumption of keeping patients in light sedation. We believe that combining our model with the *clinician-in-loop* paradigm presented by (11) may help address this issue in future works.

## Data Availability Statement

Publicly available datasets were analyzed in this study. The data can be found in: https://mimic.physionet.org/.

## Author Contributions

NE, TA, and MG contributed to the design and implementation of the research. All authors contributed to the article and approved the submitted version.

## Conflict of Interest

The authors declare that the research was conducted in the absence of any commercial or financial relationships that could be construed as a potential conflict of interest.
